# Physician–patient communication affects patient satisfaction in treatment decision-making: a structural equation modelling analysis of a web-based survey in patients with ulcerative colitis

**DOI:** 10.1007/s00535-021-01811-1

**Published:** 2021-07-27

**Authors:** Katsuyoshi Matsuoka, Hirono Ishikawa, Takeo Nakayama, Yusuke Honzawa, Atsuo Maemoto, Fumihito Hirai, Fumiaki Ueno, Noriko Sato, Yutaka Susuta, Toshifumi Hibi

**Affiliations:** 1 Division of Gastroenterology and Hepatology, Department of Internal Medicine, Toho University Sakura Medical Center, Sakura, Japan; 2 Teikyo University Graduate School of Public Health, Tokyo, Japan; 3 Department of Health Informatics, Kyoto University School of Public Health, Kyoto, Japan; 4 Department of Gastroenterology and Hepatology, Graduate School of Medicine, Kyoto University, Kyoto, Japan; 5 IBD Center, Sapporo Higashi Tokushukai Hospital, Sapporo, Japan; 6 Department of Gastroenterology and Medicine, Fukuoka University Faculty of Medicine, Fukuoka, Japan; 7 Center for Gastroenterology and Inflammatory Bowel Disease, Ofuna Chuo Hospital, Kamakura, Japan; 8 Ikuyaku. Integrated Value Development Division, Mitsubishi Tanabe Pharma Corporation, Tokyo, Japan; 9 Center for Advanced IBD Research and Treatment, Kitasato University Kitasato Institute Hospital, Kitasato University, 5-9-1 Shirokane, Minato-ku, Tokyo, 108-8642 Japan

**Keywords:** Ulcerative colitis, Physician–patient communication, Patient satisfaction

## Abstract

**Background:**

The relationship of bidirectional sharing of information between physicians and patients to patient satisfaction with treatment decision-making for ulcerative colitis (UC) has not been examined. Here, we conducted a web-based survey to evaluate this relationship.

**Methods:**

Patients aged ≥ 20 years with UC were recruited from the IBD Patient Panel and Japanese IBD Patient Association. Patients completed our web-based survey between 11 May and 1 June 2020. The main outcomes were patient satisfaction (assessed by the Decision Regret Scale) and patient trust in physicians (assessed by the Trust in Physician Scale).

**Results:**

In this study (*n* = 457), a structural equation modelling analysis showed that physician-to-patient and patient-to-physician information significantly affected patient satisfaction with treatment decision-making (standardised path coefficient: 0.426 and 0.135, respectively) and patient trust in physicians (0.587 and 0.158, respectively). Notably, physician-to-patient information had a greater impact. For patient satisfaction with treatment decision-making and patient trust in physicians, information on “disease” (indirect effect: 0.342 and 0.471, respectively), “treatment” (0.335 and 0.461, respectively), and “endoscopy” (0.295 and 0.407, respectively) was particularly important, and the level of this information was adequate or almost adequate. Patient-to-physician information on “anxiety and distress” (0.116 and 0.136, respectively), “intention and desire for treatment” (0.113 and 0.132, respectively), and “future expectations of life” (0.104 and 0.121, respectively) were also important for patient satisfaction with treatment decision-making and patient trust in physicians, but these concerns were not adequately communicated.

**Conclusions:**

Adequate physician–patient communication, especially physician-to-patient information, enhanced patient satisfaction with treatment decision-making for UC.

**Supplementary Information:**

The online version contains supplementary material available at 10.1007/s00535-021-01811-1.

## Introduction

Ulcerative colitis (UC) is an inflammatory bowel disease (IBD) of unknown aetiology that primarily affects the mucosa of the large intestine and often results in mucosal erosions and ulcerations [1–3]. Treatment for UC consists mainly of 5-aminosalicylic acid (5-ASA), corticosteroids, immunomodulators, and molecular targeted agents, including biologics [3–5]. As there is no cure for UC [2], physicians and patients face numerous treatment decisions during the clinical course of the disease. Treatment choices for UC are tailored to the disease activity and the extent of colonic involvement [1, 3–6]. Additionally, there are many therapeutic options to consider regarding the treatment regime, including differences in routes of drug administration, drug release properties, mechanisms of action, and dosing intervals [1, 3–6]. As the age range of patients with UC may vary widely [1, 6], it is also essential to consider patient preferences, life events, and lifestyle during the selection of treatment for UC.

Shared decision-making (SDM) is the process by which the physician and patient collaborate to select treatments or interventions that reflect the patient’s preferences, values, and circumstances [7, 8]. This strategy has received increasing attention from both patients and physicians in recent years, as it is key for patient-centred care [9]. SDM is considered to be important in the treatment of UC, as many treatment options are available, and patients suffering from UC have diverse background characteristics, including variations in demographic details, medical history, and special situations (e.g., pregnancy, geriatric/paediatric generations, and those with comorbidities). Recent studies showed that most patients with IBD prefer to participate in the decision-making process to select a treatment plan [10–12]. It has also been reported that IBD patient involvement in SDM is associated with treatment satisfaction [12, 13].

In SDM, it is important that patients and physicians share “information, goals, and responsibilities”, and that both parties reach a consensus and agreement as to which treatment will be implemented [7, 9, 14]. Insufficient information for patients with IBD has been reported as one of the factors affecting patient satisfaction [15]. While there have been reports that the majority of IBD patients are satisfied with communication with their physicians [16, 17], there have also been reports of a lack of information on, for example, disease course, treatments and their risks, or the patient’s social and working rights [18–20], as well as insufficient sharing of treatment goals [17, 21]. However, these were descriptive reports, and in addition, the impact of communication flow (both physician-to-patient and patient-to-physician) on patient satisfaction has not been examined.

We administered a web-based survey of UC patients to investigate the impact of physician-to-patient and patient-to-physician information on patient satisfaction with treatment decision-making and patient trust in physicians, and analysed the survey data using structural equation modelling as a multivariate statistical method. In addition, we explored the differences of these impacts by patient-background characteristics and type of information. Patient satisfaction and patient trust in physicians were quantified using two validated scales: the Decision Regret Scale (DRS) [22] and the Trust in Physician Scale (TIPS). [23].

## Methods

### Study design

This cross-sectional study was conducted using an anonymous web-based questionnaire in patients with UC. The survey period was between 11 May 2020 and 1 June 2020. The study was registered in the University Hospital Medical Information Network (UMIN000040343).

Participants were recruited from the IBD Patient Panel developed and maintained by QLife, Inc. (Tokyo, Japan) and the Japanese IBD Patient Association. Patients provided informed consent on the respective survey study website before answering the survey questionnaire. Inclusion criteria were age ≥ 20 years, diagnosis of UC, regular attendance at a medical institution (at least once every 3 months, the standard visit frequency in Japan) for UC treatment, and agreement to participate and informed consent provision on the survey study website. There were no specific exclusion criteria.

This web-based survey complied with the Declaration of Helsinki of the World Medical Association (amended October 2013) and the Ethical Guidelines for Medical Research Involving Human Subjects (partially amended on 28 February 2017). The Takahashi Clinic Institutional Review Board approved the study protocol and related documents on 21 April 2020.

### Study endpoints

The primary endpoint was the impact of physician-to-patient and patient-to-physician information on patient satisfaction with treatment decision-making. Secondary endpoints were the impact of physician-to-patient and patient-to-physician information on patient trust in physicians, and the impacts of patient-background characteristics and the types of information being provided. Participants were required to answer the survey based on memory recall regarding treatment decision-making at the time of the most recent UC relapse (initial diagnosis if no relapse had occurred).

### Survey items

#### Patient background

Patient demographics and clinical characteristics, including age, sex, time since UC diagnosis, current symptoms based on the two-item patient-reported outcome tool (PRO2 [24]; where remission was defined as bleeding = 0 and stool frequency ≤ 1), and therapeutic agents received (at the time of UC relapse and present), were recorded. In addition, details relating to the medical institution at which treatment decision-making took place, time spent with the physician (when deciding on treatment, routine clinical care), and patient decision-making preference score based on the four general items of the Autonomy Preference Index and rated on a five-point scale [25] (where 1 = lowest decision-making preference) were captured.

#### Physician–patient communication at the time of treatment decision-making

“Physician-to-patient information” was defined as information provided by a physician to a patient regarding disease (e.g., overall disease course and future outcomes), endoscopy (e.g., characteristics, significance, safety, time taken for examination), treatment (e.g., characteristics, safety, effectiveness), medical costs for UC treatment (e.g., medical costs for UC treatment, specific medical expenses), support (e.g., medical cost consultation by social workers, employment support by human resources companies, public medical support), and precautions for daily life (exercise, diet, and travel, among others). Each item was rated on a four-point scale as (1) adequate; (2) almost adequate; (3) somewhat inadequate; and (4) inadequate.

“Patient-to-physician information” included information provided by a patient to a physician regarding UC symptoms (stool frequency, presence of bloody stool, abdominal pain, and diarrhoea, among others), general understanding of the disease (e.g., overall illness course, future outcome), anxiety and distress at the time of treatment decision-making, intention and desire for treatment, and future expectations of life. The level of assessment was classified in four stages: (1) fully communicated; (2) somewhat communicated; (3) poorly communicated; and (4) never communicated. “Physician-to-patient information” and “patient-to-physician information” items were selected based on the roles of physicians and patients required by SDM and previous reports [16, 18–20].

#### Patient satisfaction with treatment decision-making and patient trust in physicians

DRS [22] was used to investigate patient satisfaction with the treatment decision. DRS is composed of five items and is evaluated based on a five-point score, in which 1 means “strongly agree” and 5 means “strongly disagree”. The final score is then converted to a scale of 0–100, and the resulting score reflects the degree of patient satisfaction regarding the determination of the treatment plan. The score cut-off for no/mild regret was set at 25 points. [26].

Patient trust in their physicians was evaluated using TIPS [23]. TIPS is composed of 11 items and is evaluated based on a five-point score, in which 1 means “strongly disagree” and 5 means “strongly agree”. The final score is then converted to a scale of 0–100. The resulting score reflects the extent to which the patient trusts their physician.

### Statistical analysis

Assuming a population size of approximately 200,000 Japanese UC patients, with a 95% confidence (*z* score = 1.96, Type I error, a significant level of 0.05), an allowable error of 5% (*e* = 0.05, Type II error, a detection power of 95%), and a response ratio (proportion of respondents for a given response) of 50% (*p* = 0.50), the estimated sample size was 384 cases. In addition, accounting for possible dropouts before completing the survey and exclusions due to duplicate responses, the sample size was set at 500 patients. After excluding duplicate respondents and those who voluntarily withdrew consent after completion of the web-based survey, all patients who completed the survey were included in the analysis.

Hypothetical models for the primary and secondary endpoints were generated (Supplementary Fig. 1) and analysed by structural equation modelling. A confirmatory factor analysis was carried out by the data of physician–patient communication to determine the latent variables of “physician-to-patient information” and “patient-to-physician information”. Correlations between patient-background characteristics and DRS or TIPS were examined using the Pearson’s correlation coefficient, and variables that were significantly correlated (with an absolute correlation coefficient of 0.2 or greater) were added to the path diagram. The relationships between DRS or TIPS and categorical patient-background characteristics were evaluated by analysis of variance (ANOVA), and multi-population simultaneous analyses were conducted using variables that showed a significant difference. The structural equation model was constructed using data obtained by reversing the five-point scale of TIPS.

To identify the information that patients considered important but insufficient, we created a correlation chart of the indirect effects of each information item (the value multiplied by the standardised path coefficient via the observed variables of information, the latent variables of information, and DRS or TIPS) in the structural equation model and the proportions of patients who answered “somewhat inadequate or inadequate” and “poorly communicated or never communicated” regarding each information item.

Continuous variables were presented as mean and standard deviation (SD) or median and interquartile range (IQR), and categorical variables as frequency and percentage. A *p* value of < 0.05 was considered statistically significant. The analysis software used for multivariate analysis was developed by Eystat Co., Ltd., Tokyo, Japan. Additionally, Excel (Microsoft Co., USA), Excel statistics for covariance structure-analysis Ver.2.0 (Esmi Co., Ltd., Japan), and IBM SPSS Amos version 25 (IBM Corp., Armonk, NY, USA; SPSS Inc., Chicago, IL, USA; and Amos Development Corp, Medville, PA, USA) were used.

## Results

### Patients

During the survey period, 472 patients with UC completed the questionnaire. Fifteen duplicate responses were excluded, and 457 were included in this analysis. The male-to-female ratio was 4:6 (Table 1). Patients had a mean (SD) age and time since diagnosis of 43.1 (12.5) years and 8.3 (8.5) years, respectively. The mean (SD) PRO2 score was 1.4 (1.4), and the percentage of patients with PRO2 remission was 57.5%. The mean (SD) and median (IQR) patient decision-making preference scale scores were 2.6 (0.4) and 2.5 (2.3–2.8), respectively.

**Table 1:** Patient demographic and clinical characteristics

	*n* = 457	
Sex		
Male, *n* (%)	172 (37.6)	
Female, *n* (%)	285 (62.4)	
Age, years (mean ± SD)	43.1 ± 12.5	
Time since diagnosis, years (mean ± SD)	8.3 ± 8.5	
Current symptoms^a^		
PRO2 score (mean ± SD)	1.4 ± 1.4	
PRO2 remission^b^ [*n* (%)]	263 (57.5)	
Therapeutic drug	Most recent UC relapse	Current
5-ASA (enema/suppository) [*n* (%)]	135 (29.5)	117 (25.6)
5-ASA (oral) [*n* (%)]	253 (55.4)	371 (81.2)
Corticosteroid (enema) [*n* (%)]	144 (31.5)	71 (15.5)
Corticosteroid (oral) [*n* (%)]	167 (36.5)	59 (12.9)
Corticosteroid (IV) [*n* (%)]	49 (10.7)	4 (0.9)
Immunomodulator (oral) [*n* (%)]	73 (16.0)	85 (18.6)
Immunomodulator (IV) [*n* (%)]	6 (1.3)	0 (0)
Biologic agent [*n* (%)]	90 (19.7)	124 (27.1)
JAK inhibitor [*n* (%)]	14 (3.1)	12 (2.6)
Other [*n* (%)]	42 (9.2)	53 (11.6)
Healthcare institutions where the treatment plan was selected at the time of UC relapse		
National and public hospitals [*n* (%)]	105 (23.0)	
University hospital [*n* (%)]	132 (28.9)	
Clinic [*n* (%)]	81 (17.7)	
Other^c^ [*n* (%)]	139 (30.4)	
Time spent with the physician, min	When deciding on treatment	During routine clinical care
< 5 [*n* (%)]	47 (10.3)	112 (24.5)
5 to < 10 [*n* (%)]	147 (32.2)	208 (45.5)
10 to < 15 [*n* (%)]	133 (29.1)	96 (21.0)
15 to < 30 [*n* (%)]	73 (16.0)	26 (5.7)
≥ 30 [*n* (%)]	57 (12.5)	15 (3.3)
Patient decision-making preference scale (mean ± SD)	2.6 ± 0.4	

*ASA* aminosalicylate, *IV* intravenous, *JAK* Janus kinase, *PRO2* two-item patient-reported outcomes, *UC* ulcerative colitis

^a^Symptoms in the last 3 days

^b^PRO2 remission was defined as bleeding = 0 and stool frequency ≤ 1

^c^Hospital other than a national and public hospital or university hospital

Among the surveyed population, 55.4% and 29.5% of patients received oral and enema/suppository 5-ASAs, respectively; 36.5%, 31.5%, and 10.7% of patients received corticosteroids in oral, enema and IV presentations, respectively; 16.0% had received an oral immunomodulator, such as azathioprine; and 19.7% were prescribed biologics during their most recent UC relapse. The most common amount of time spent with the physician when deciding on treatment was ≥ 5 to < 10 min in 32.2% of patients, followed by ≥ 10 to < 15 min in 29.1%, and ≥ 15 to < 30 min in 16.0%.

### Physician–patient communication at the time of treatment decision-making

**Fig. 1:**
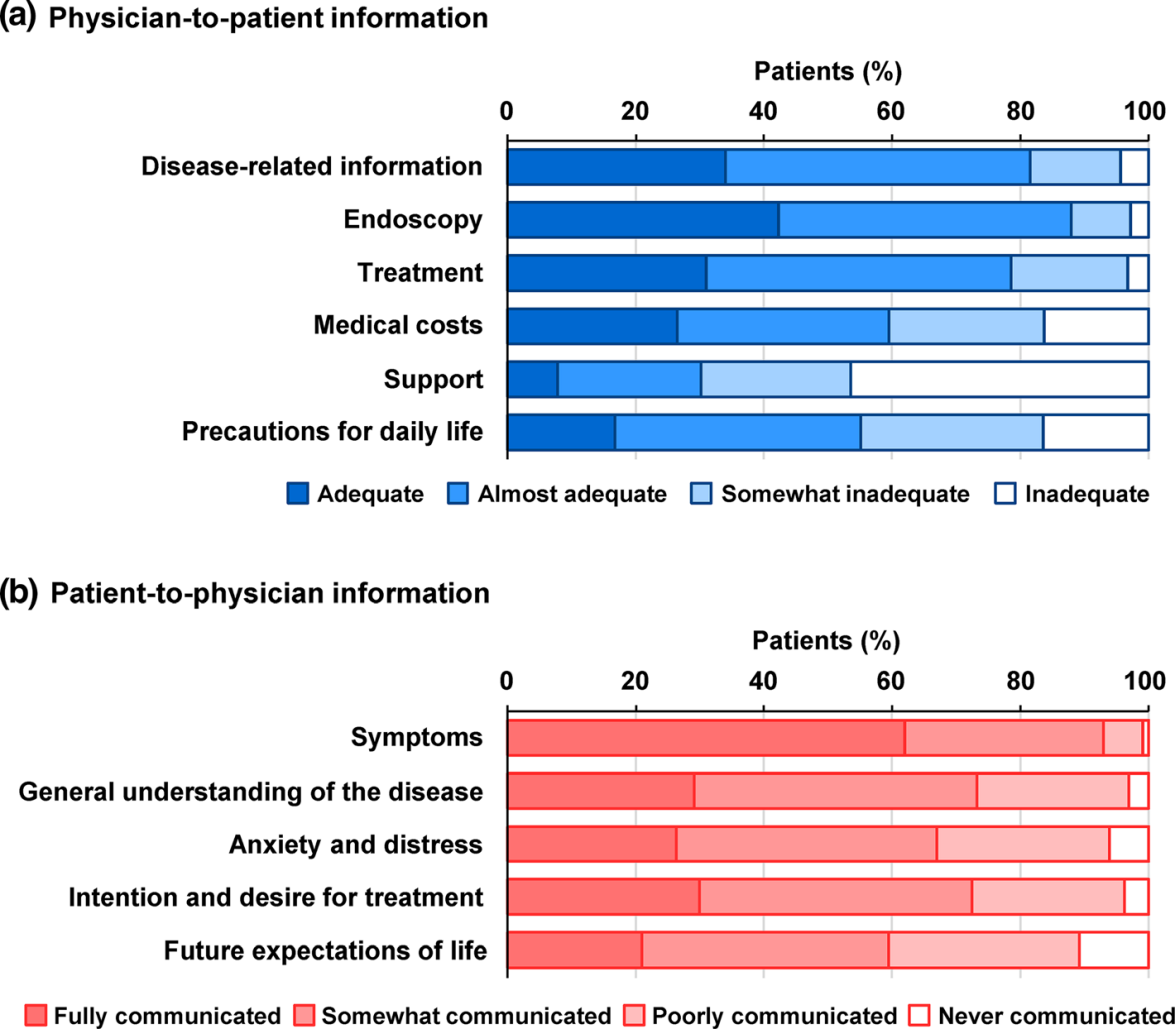
Physician–patient communication at the time of treatment decision-making. **a** Physician-to-patient information. **b** Patient-to-physician information

Regarding physician-to-patient information (Fig. 1a), patients tended to receive adequate or almost adequate information on endoscopy (88.0%), disease (81.6%), and treatment (78.6%). Patients considered that the information was inadequate mainly for support (46.4%), which includes information on medical cost consultation by social workers and employment support, followed by precautions for daily life (16.4%) and information on medical costs (16.2%).

Regarding patient-to-physician information (Fig. 1b), a large proportion of patients (93.0%) felt that they were able to fully or somewhat communicate with their physicians regarding symptoms, followed by general understanding of the disease (73.3%) and intention and desire for treatment (72.4%).

### Patient satisfaction with the treatment decision-making and patient trust in physicians

**Fig. 2:**
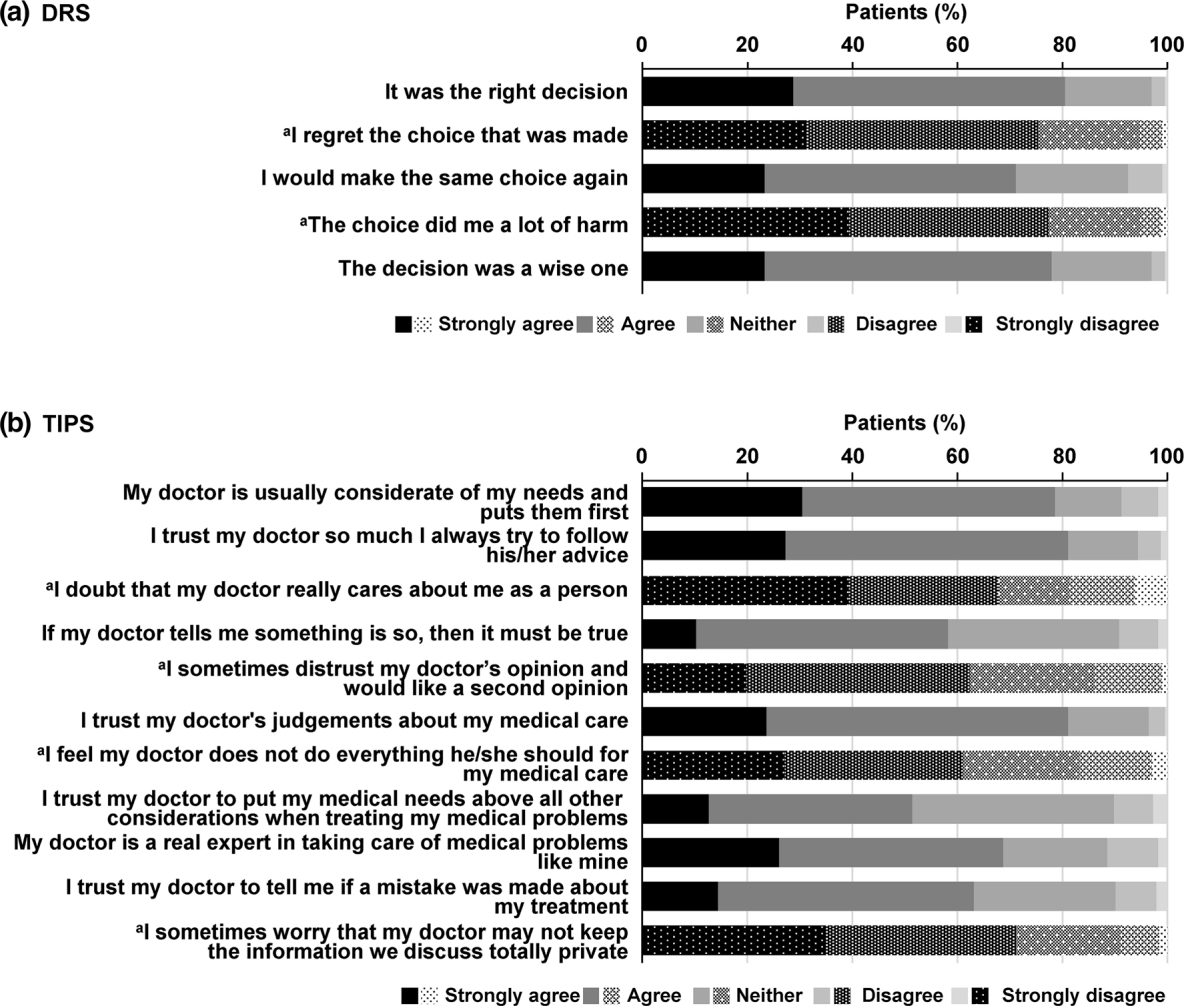
Patient satisfaction with treatment decision-making and patient trust in physicians. **a** DRS. **b** TIPS. ^a^Reversal items. *DRS* Decision Regret Scale, *TIPS* Trust in Physician Scale

Figure 2a and b show the results of the DRS and TIPS items, which reflect patient satisfaction with the treatment decision-making and patient trust in physicians. The mean (SD) DRS was 25.0 (17.3) and the percentage of individuals classified as “no/mild regret” (DRS of 25 or less) was 62.6% (286/457). The mean (SD) TIPS was 69.8 (17.1).

### Relationship between patient satisfaction with treatment decision-making or patient trust in physicians and patient background

Background characteristics that correlated with DRS or TIPS (absolute correlation coefficient of ≥ 0.2) were PRO2 score, time spent with the physician when deciding on treatment, and patient decision-making preference scale (Table 2). Patient-background characteristics that were significantly related to DRS and TIPS were current PRO2 remission/non-remission, time spent with the physician when deciding on treatment (< 10 min/10 to < 15 min/ ≥ 15 min), and patient decision-making preference scale score (≤ 2.50 [lower patient decision-making preference]/ ≥ 2.75 [higher patient decision-making preference]) (Table 2).

**Table 2:** Background characteristics correlating with DRS or TIPS

Item	DRS			TIPS		
	Correlation coefficient^a^	*p*-value^a^	*p*-value^b^	Correlation coefficient^a^	*p*-value^a^	*p*-value^b^
Sex	− 0.037	0.424	0.424	0.048	0.305	0.305
Age, years	0.089	0.059	–	− 0.016	0.735	–
20–34 years, 35–49 years, ≥ 50 years	–	–	0.063	–	–	0.962
Time since diagnosis, years	0.104	0.026	–	− 0.033	0.476	–
≤ 3 years, 4–9 years, ≥ 10 years	–	–	0.225	–	–	0.306
PRO2 score	0.262	< 0.001	–	− 0.087	0.064	–
PRO2 remission, non-remission	–	–	< 0.001	–	–	0.008
Time spent with the physician when deciding on treatment	− 0.167	< 0.001	–	0.252	< 0.001	–
< 10 min, 10 to < 15 min, ≥ 15 min	–	–	0.001	–	–	< 0.001
Patient decision making preference scale	0.215	< 0.001	–	− 0.215	< 0.001	–
≤ 2.50^c^, ≥ 2.75	–	–	< 0.001	–	–	< 0.001

*ANOVA* analysis of variance, *DRS* Decision Regret Scale, *PRO2* two-item patient-reported outcomes, *TIPS* Trust in Physician Scale, *UC* ulcerative colitis

^a^Pearson’s correlation coefficient

^b^ANOVA

^c^Median

### Impact of physician–patient communication on patient satisfaction with treatment decision-making and patient trust in physicians

**Fig. 3:**
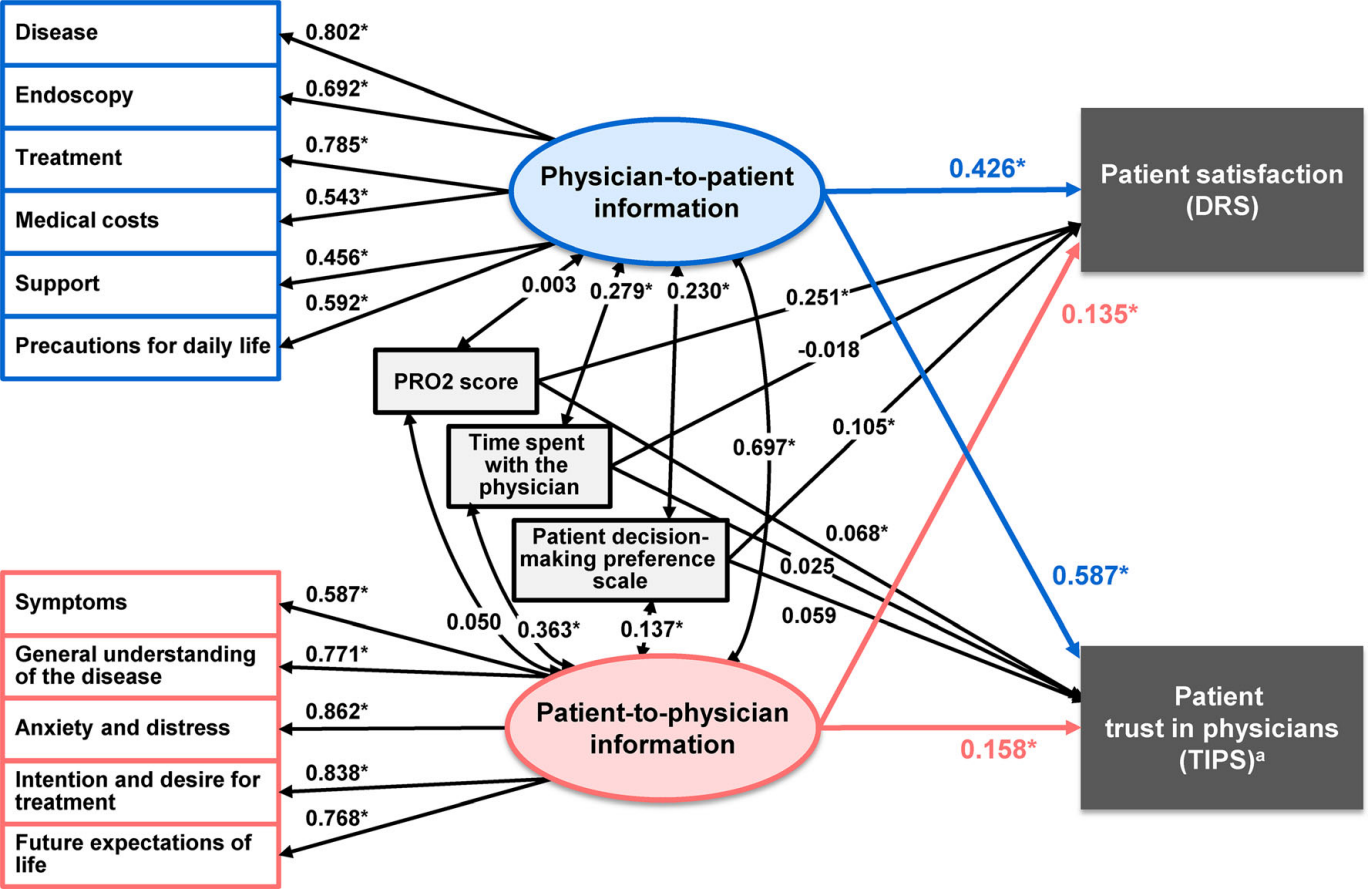
Path diagram in the structural equation modelling showing the relationship between physician–patient communication, patient satisfaction with treatment decision-making, and patient trust in physicians. The GFI, AGFI, and RMSEA of the path diagram, including patient-background correlated with DRS or TIPS with an absolute correlation coefficient of ≥ 0.2 were 0.908, 0.860, and 0.080, respectively. Values represent standardized path coefficients that indicate the degree of relationship between variables. *p < 0.05. ^a^Data reversing the five-level rank values of TIPS. *AGFI* adjusted goodness-of-fit index, *DRS* Decision Regret Scale, *GFI* Goodness-of-fit index, *PRO2* two-item patient-reported outcomes, *RMSEA* root mean square error of approximation, *TIPS* Trust in Physician Scale

Patient-background factors (PRO2 score, time spent with the physician when deciding on treatment, and patient decision-making preference scale) correlated with DRS or TIPS with an absolute correlation coefficient of ≥ 0.2 were included in the path diagram. The goodness-of-fit index, adjusted goodness-of-fit index, and root mean square error of approximation were 0.908, 0.860, and 0.080, respectively, showing statistically acceptable goodness of fit (Fig. 3). Physician-to-patient information significantly affected patient satisfaction with treatment decision-making (standardised path coefficient: 0.426). Patient-to-physician information also significantly affected patient satisfaction with the treatment decision-making (0.135), although the impact was smaller than that of physician-to-patient information. Among patient-background characteristics, current PRO2 scores affected patient satisfaction with treatment decision-making (0.251). Physician–patient communication also significantly affected patient trust in physicians, with physician-to-patient information (0.587) having a greater impact than patient-to-physician information (0.158). Neither PRO2 score, time spent with the physician when deciding on treatment, nor the patient decision-making preference scale significantly affected patient trust in physicians.

The standardised path coefficients from physician-to-patient information (latent variable) to each item were all above 0.4. Among these items, the highest standardised path coefficients were those for disease-related (0.802) and treatment-related (0.785) information (Fig. 3). Standardised path coefficients from patient-to-physician information as latent variable to each item were all above 0.5. Among these, the highest standardised path coefficients were those for information on anxiety and distress (0.862) and intention and desire for treatment (0.838) (Fig. 3).

A correlation was observed between the physician-to-patient information score and the patient-to-physician information score at the time of treatment decision-making (0.697) (Fig. 3). The time spent with the physician and patient decision-making preference scale also correlated with the physician-to-patient information score (0.279 and 0.230, respectively) and patient-to-physician information score (0.363 and 0.137, respectively).

### Results of multi-population simultaneous analyses by categorical variables of patient background

Multi-population simultaneous analyses by categorical patient-background variables were applied in the path diagram (Supplementary Fig. 1). Physician-to-patient information had a high impact on patient satisfaction with treatment decision-making and patient trust in physicians in both PRO2 remission (standardised path coefficient: 0.489 and 0.567, respectively) and non-remission (0.475 and 0.756, respectively) groups (Supplementary Fig. 2a). The patient-to-physician information tended to affect patient satisfaction with treatment decision-making in the non-remission group (0.163) and affected patient trust in physicians in the remission group (0.206).

In multi-population simultaneous analyses by the time spent with physicians when deciding on treatment (< 10 min, 10 to < 15 min, and ≥ 15 min), physician-to-patient information had a high impact on patient satisfaction with treatment decision-making (0.526, 0.309, and 0.416, respectively) and patient trust in physicians (0.553, 0.602, and 0.655, respectively) in all groups (Supplementary Fig. 2b). The patient-to-physician information did not affect patient satisfaction with treatment decision-making in the group with less than 10 min spent with physicians when deciding on treatment (0.069) but had a higher impact on patient trust in physicians in the group with the shorter time spent with physicians when deciding on treatment (0.245).

In a multi-population simultaneous analysis by patient decision-making preference scale (≤ 2.50 and ≥ 2.75), physician-to-patient information affected patient satisfaction with treatment decision-making (0.220 and 0.661, respectively) and patient trust in physicians (0.538 and 0.665, respectively) in both groups (Supplementary Fig. 2c). The impact of physician-to-patient information on patient satisfaction was higher in the group with higher patient decision-making preference. The patient-to-physician information affected patient satisfaction with treatment decision-making (0.269) and patient trust in physicians (0.197) in the group with the lower patient decision-making preference.

### Information that patients considered important but insufficient

**Fig. 4:**
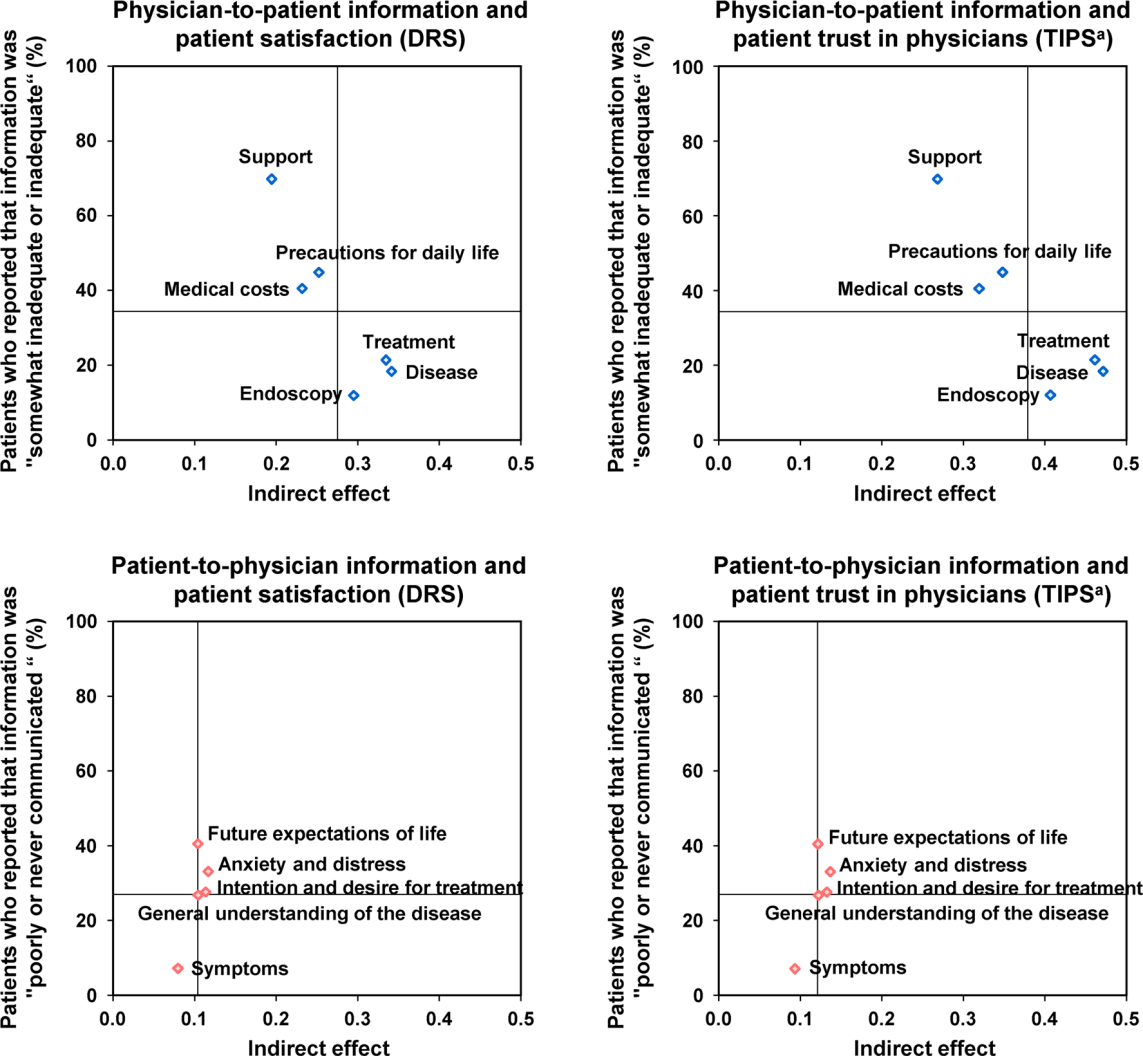
Correlation diagram between indirect effects of individual information items in the structural equation model and the proportions of patients who reported that information was “somewhat inadequate or inadequate” and information was “poorly communicated or never communicated”. Indirect effects were the value multiplied by the standardised path coefficient via the observed variables of information, the latent variables of information, and DRS or TIPS in the structural equation model. The lines show the mean value for each axis. ^a^Data reversing the five-level rank values of TIPS. *DRS* Decision Regret Scale, *TIPS* Trust in Physician Scale

Correlation plots between the indirect effects of each information item and the proportions of patients who reported that information was “somewhat inadequate or inadequate” and information was “poorly communicated or never communicated” indicated that among the physician-to-patient information, “disease” (indirect effect: 0.342 and 0.471, respectively), “treatment” (0.335 and 0.461, respectively), and “endoscopy” (0.295 and 0.407, respectively) were important for patient satisfaction with treatment decision-making as well as patient trust in physicians, and the information on these was adequate or almost adequate (Fig. 4). Among the patient-to-physician information, “anxiety and distress” (0.116 and 0.136, respectively), “intention and desire for treatment” (0.113 and 0.132, respectively), and “future expectations of life” (0.104 and 0.121, respectively) were important for patient satisfaction with treatment decision-making as well as patient trust in physicians, as shown in Fig. 3, but information on these was lacking (Fig. 4).

## Discussion

The results of this web-based survey in patients with UC using a structural equation modelling analysis showed that physician–patient communication affected patient satisfaction with treatment decision-making and patient trust in physicians. Comparing the information exchange from physicians to patients and patients to physicians, the physician-to-patient information had a greater impact on patient satisfaction with treatment decision-making. The present analysis indicated that information on “disease” (e.g., overall disease course and future outcomes), “treatment” (e.g., characteristics, safety, and effectiveness), and “endoscopy” (e.g., characteristics, significance, safety, and time taken for examination) were particularly important for patient satisfaction with treatment decision-making and patient trust in physicians. The level of information on these was adequate or almost adequate, suggesting that physicians and patients recognized the importance of sharing this information. Although patients regarded patient-to-physician information on “anxiety and distress”, “intention and desire for treatment”, and “future expectations of life” as important items for patient satisfaction with treatment decision-making and developing trust in physicians, they felt that this information had not been fully communicated with their physician.

In this survey, relatively few patients were prescribed immunomodulators, biologics, or the Janus kinase inhibitor at the time of the most recent UC relapse. Additionally, the mean current PRO2 score was 1.4, and approximately 60% of patients were in PRO2 remission. From these data, we can infer that the survey respondents may have had low symptom severity.

We found that high proportions of physician-to-patient information items and patient-to-physician information items were described as “adequate or almost adequate” and “fully communicated or somewhat communicated”, respectively. These results are consistent with some prior studies [16, 17]. Although a high proportion of respondents indicated that the physician-to-patient information on “support” (such as medical cost consultation by social workers, employment support by human resources companies, and public medical support) was inadequate, it is possible that information on “support” was to be communicated by medical staff other than physicians, though we did not have data to assess these other patient-provider dyads. Patient satisfaction with the treatment decision-making and patient trust in physicians were also relatively high. It has previously been reported that the therapeutic effectiveness of a drug was the attribute most frequently considered important or very important by patients [27]. Therefore, our results may be attributable to the fact that many patients in this survey had relatively stable symptoms and were not difficult-to-treat.

Respondents of this survey valued physician-to-patient information about “disease” and “treatment” for patient satisfaction with treatment decision-making and patient trust in physicians. The information on “support”, which was found to be insufficient in this survey, also affected patient satisfaction with treatment decision-making and patient trust in physicians. The results of the multi-population simultaneous analysis showed that physician-to-patient information was of great importance in all groups, regardless of background characteristics. However, physician-to-patient information more strongly affected patient satisfaction with treatment decision-making and patient trust in physicians in the group with the higher patient decision-making preference than the group with the lower decision-making preference. These results indicate that patients who prefer to be involved in treatment decision-making wish to obtain more information from their physicians.

Patient-to-physician information had a significant impact on patient satisfaction with treatment decision-making and patient trust in physicians, but the impact was lower compared with the physician-to-patient information. These results may suggest that patients believe it is more important to obtain information from physicians than to communicate information themselves. Although an SDM approach may be preferred by some patients, other patients may prefer to leave the important treatment decisions to the physician [28]. In addition, it has been reported that physicians may exert control of communication during consultations by asking more questions than the patients [29]. In analyses by current PRO2 remission and non-remission groups, the patient-to-physician information items tended to affect patient satisfaction with treatment decision-making in the non-remission group. Based on these results, the fact that the study population included many patients with stable symptoms is also considered one of the reasons for the low impact of patient-to-physician information items. In the group with low patient decision-making preference, patient-to-physician information still affected patient satisfaction with treatment decision-making and patient trust in physicians. Despite low decision-making preferences, it remains important for these patients to communicate their symptoms, preferences, and needs to their physicians so that their physicians can make better decisions for them.

In recent years, SDM has gained attention as a method for consensus-building between physicians and patients. The process of SDM is bidirectional and interactive. Important factors are agreement processes on a treatment plan, building a relationship between patients and healthcare professionals, and ensuring that the outcomes align with the quality of life goals of patients [7]. Several studies in IBD populations have shown that patients who were involved in SDM reported higher treatment satisfaction than those who were not engaged in SDM. [12, 13] In this study, we found that patients felt that the patient-to-physician information on “anxiety and distress”, “intention and desire for treatment”, and “future expectations of life” were important for patient satisfaction with treatment decision-making and patient trust in physicians. Despite this, patients had not been able to communicate fully with their physicians regarding these topics. Patients with IBD have disability, including not only IBD-related factors but also psychological and social factors, and it has been shown that more than 30% of patients may disclose their disability even during clinical remission [30]. However, it has been reported that approximately half of UC patients were unable to discuss adequately with their physicians about their quality of life [31] and emotional concerns [17]. Stress management has been reported to produce important clinical benefit in patients with rheumatoid arthritis or asthma [32, 33]. Therefore, in clinical practice, patient-to-physician information on “anxiety and distress”, “intention and desire for treatment”, and “future expectations of life” is considered to be clinically meaningful. These results indicate that implementing interactive SDM by increasing patient-to-physician communication may further enhance patient satisfaction with treatment decision-making and patient trust in physicians. Rubin et al. have reported that top-ranked resources that physicians considered helpful in improving patient communication with UC patients were online tools or smartphone applications [17]. Information leaflets for patients have been shown to improve physician–patient communication, although the study addressed another disease [34]. In the future, it will be necessary to further promote physician–patient communication by considering the utilization of tools to improve communication and by increasing patient awareness that more in-depth patient-to-physician communication can lead to better treatment outcomes and patient satisfaction. This is the first study to show the difference in the impact of physician-to-patient information and patient-to-physician information on patient satisfaction with treatment decision-making and patient trust in physicians. In the future, it will be necessary to examine how clinically significant this difference is, and whether differences in the impact of physician-to-patient information and patient-to-physician information on patient satisfaction with treatment decision-making are reduced by the adequate role of physicians and patients in SDM.

This study had some limitations. First, all responses to the questionnaire were self-reported by patients and consisted primarily of their retrospective assessment of physician communications and treatment decision-making. This likely involved a degree of recall bias and adversely affected data reliability. Second, physician’s characteristics, such as sex and age, were not included in the questionnaires. Therefore, the present results do not consider physician’s background factors. Third, the data were obtained via a web-based survey, meaning that the population was limited to those who had access to the web site. The age distribution of the analysed population did not differ significantly from that of adult UC patients in Japan [35], although there were concerns about bias in age groups because elderly patients may have a lower internet usage than non-elderly patients. In addition, as a result of recruitment from the IBD Patient Panel and the Japanese IBD Patient Association, the proportion of patients who actively seek information may be high in patients registered in such a panel, and the participants may have been more likely to seek information from physicians. Finally, the proportion of females who responded was somewhat higher than the known male-to-female ratio (1.24) of UC patients in Japan [36]. However, the current study did not find a significant relationship between sex and patient satisfaction in treatment decision-making and trust in physicians. Therefore, we believe that the higher than expected proportion of women in this sample did not have a major impact on our study results.

In conclusion, this web-based survey in patients with UC revealed that adequate physician–patient communication enhanced patient satisfaction with treatment decision-making and patient trust in physicians. Notably, physician-to-patient information had a greater impact on patient satisfaction with treatment decision-making and patient trust in physicians than did patient-to-physician information. The information that patients considered important but insufficiently addressed was patient-to-physician information on “future expectations of life”, “anxiety and distress”, and “intention and desire for treatment”. Our findings may provide useful information for physician–patient communication to further enhance patient satisfaction with treatment decision-making for UC.

## Supplementary Information

Below is the link to the electronic supplementary material.

Supplementary file1 (PDF 122 KB)

## Abbreviations

5-ASA: 5-Aminosalicylic acid
AGFI: Adjusted goodness-of-fit index
ANOVA: Analysis of variance
DRS: Decision Regret Scale
GFI: Goodness-of-fit index
IBD: Inflammatory bowel disease
IQR: Interquartile range
IV: Intravenous
JAK: Janus kinase
PRO2: Two-item patient-reported outcome
RMSEA: Root mean square error of approximation
SD: Standard deviation
SDM: Shared decision-making
TIPS: Trust in Physician Scale
UC: Ulcerative colitis
