# Supplementary material for: Physician–patient communication affects patient satisfaction in treatment decision-making: a structural equation modelling analysis of a web-based survey in patients with ulcerative colitis
Source: J Gastroenterol. 2021 Jul 27;56(9):843–55. doi: 10.1007/s00535-021-01811-1 (PMC8370900; doi:10.1007/s00535-021-01811-1)

Supplementary material

Supplementary Fig. 1 Relationship between physician-patient communication, patient satisfaction with the treatment decision-making, and patient trust in physicians (hypothetical model).

[a]Data reversing the five-level rank values of TIPS.

DRS Decision Regret Scale, *TIPS* Trust in Physician Score

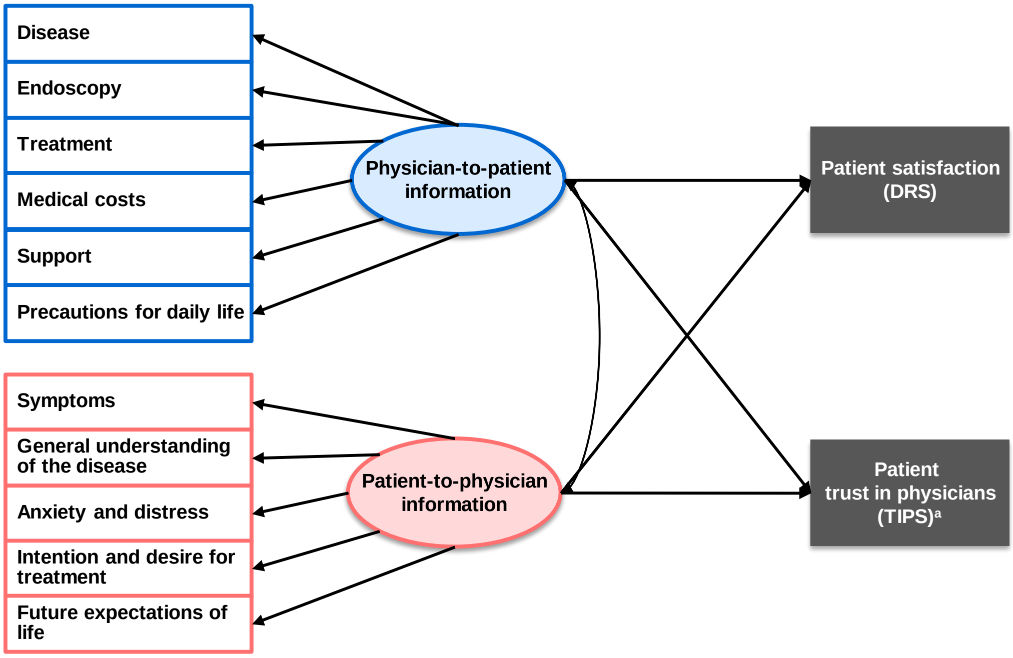

Supplementary Fig. 2 Path diagram showing the relationship between physician-patient communication, patient satisfaction with treatment decision-making, and patient trust in physicians by categorical variables of patient background (multi-population simultaneous analysis). (a) By PRO2 remission and non-remission. GFI = 0.880, AGFI = 0.817, RMSEA = 0.101. (b) By the time spent with the physician when deciding on treatment. GFI = 0.857, AGFI = 0.782, RMSEA = 0.105. (c) By the patient decision-making preference scale score. The patient decision-making preference scale was stratified by median. GFI = 0.877, AGFI = 0.814, RMSEA = 0.099. Values represent standardized path coefficients that indicate the degree of relationship between variables.

*p<0.05.

[a]Data reversing the five-level rank values of TIPS.

AGFI adjusted goodness of fit index, *DRS* Decision Regret Scale, *GFI* Goodness-of-fit index, *PRO2* two-item patient reported outcomes, *RMSEA* root mean square error of approximation, *TIPS* Trust in Physician Scale, *UC* ulcerative colitis

a. By PRO2 remission/non-remission

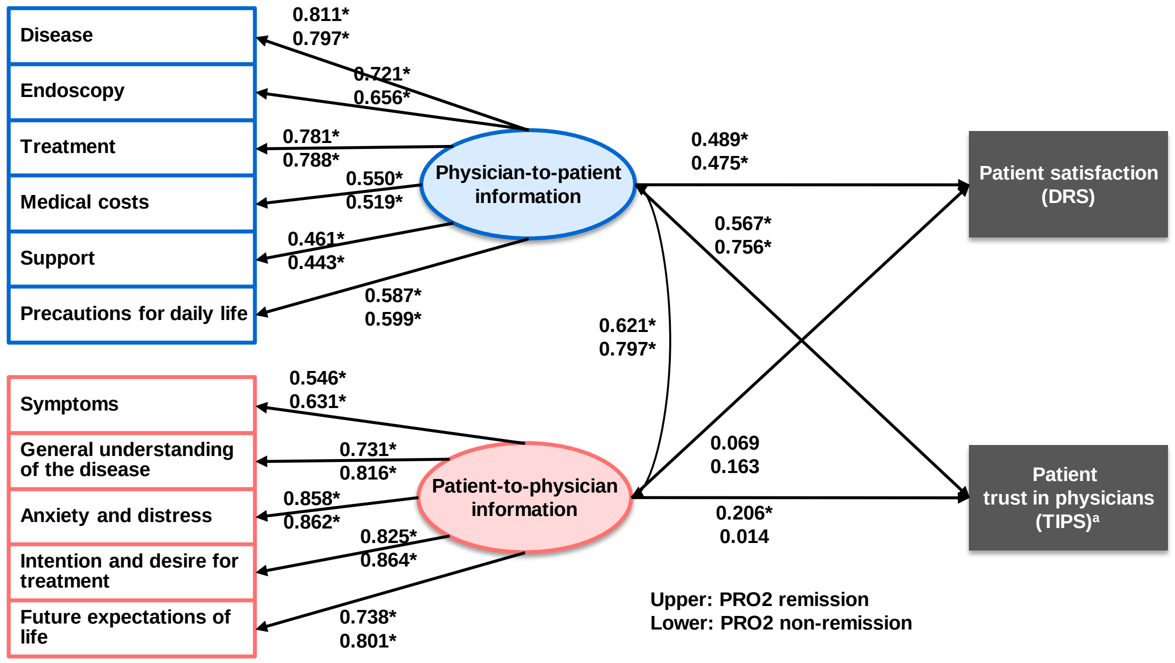

b. By the time spent with the physician when deciding on treatment

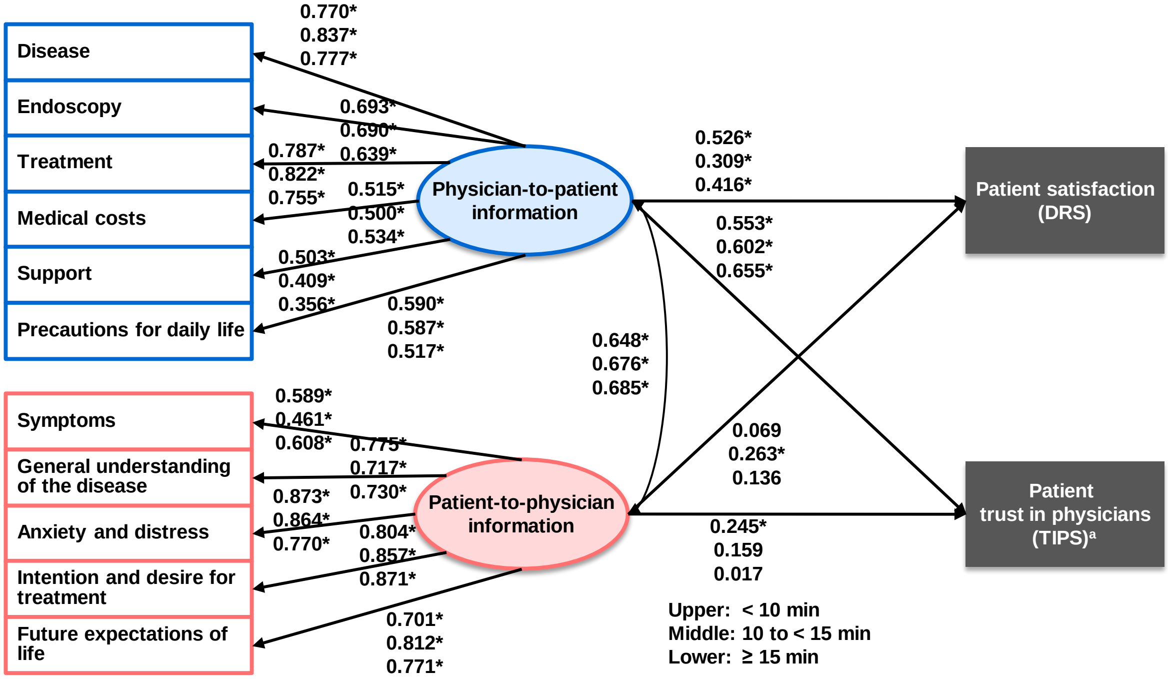

c. By patient decision-making preference scale

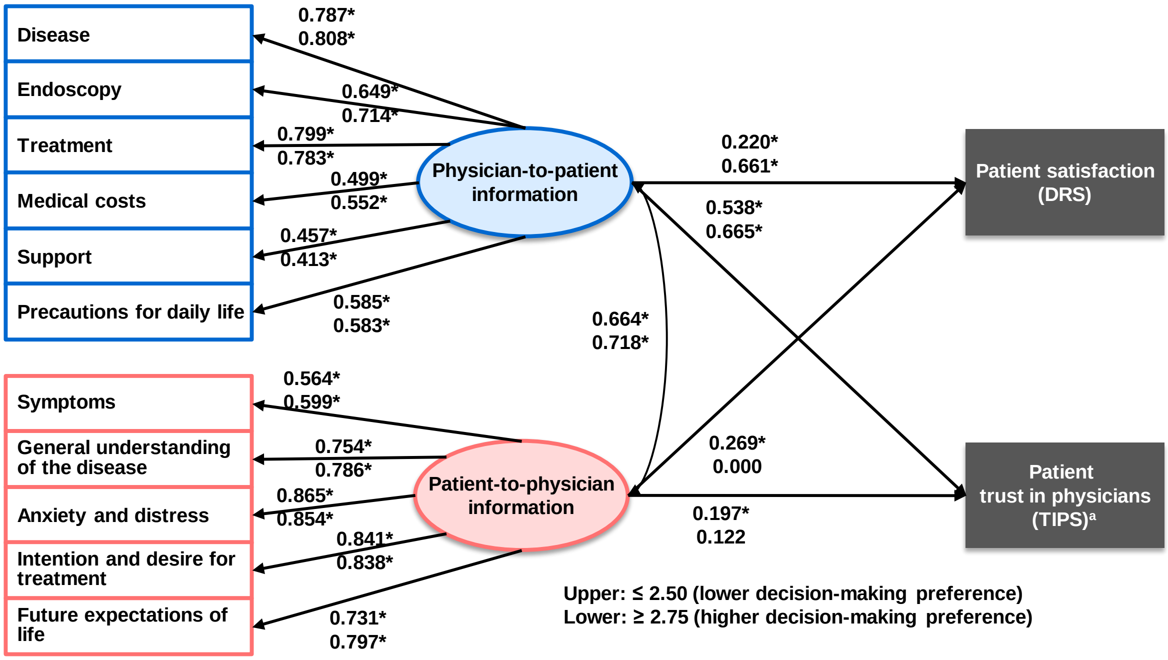

Supplement: Supplementary file 1 — Supplementary file1 (PDF 122 KB) [file 535_2021_1811_MOESM1_ESM.pdf]
